# Investigation of CD26, a potential SARS-CoV-2 receptor, as a biomarker of age and pathology

**DOI:** 10.1042/BSR20203092

**Published:** 2020-11-26

**Authors:** Animesh Alexander Raha, Subhojit Chakraborty, James Henderson, Elizabeta Mukaetova-Ladinska, Shahid Zaman, John Trowsdale, Ruma Raha-Chowdhury

**Affiliations:** 1 John van Geest Centre for Brain Repair, Department of Clinical Neuroscience, University of Cambridge, Cambridge, U.K.; 2 CamKolInv, Cambridge Kolkata Innovation and Therapeutic Limited, Cambridge, U.K.; 3 Department of Neuroscience, Psychology and Behaviour, University of Leicester, Leicester, U.K.; 4 Department of Psychiatry, Cambridge Intellectual and Developmental Disabilities Research Group, Cambridge, U.K.; 5 Division of Immunology, Department of Pathology, University of Cambridge, Cambridge, U.K.

**Keywords:** age-related dementia, Corona virus COVID-19, Covid-19 biomarker, dipeptidyl peptidase 4 (DPP4), SARS-CoV-2 infection, Type 2 diabetes mellitus

## Abstract

**Objective:** In some individuals, coronavirus severe acute respiratory syndrome coronavirus-2 (SARS-CoV-2) infection leads to a variety of serious inflammatory symptoms, including blood clotting and acute respiratory distress. Death due to COVID-19 shows a steep rise in relation to age. Comorbidities such as type 2 diabetes mellitus (T2DM), hypertension, and cardiovascular disease also increase susceptibility. It has been reported that T-cell regulatory dipeptidyl peptidase 4 (DPP4; cluster of differentiation 26 (CD26)) binds to the external spike (S) glycoprotein of SARS-CoV-2 as a receptor, for the viral entry into the host cell. CD26 is expressed on many cells, including T and natural killer (NK) cells of the immune system, as a membrane-anchored form. A soluble form (sCD26) is also found in the blood plasma and cerebrospinal fluid (CSF).

**Approach and results:** To investigate a possible relationship between sCD26 levels, age and pathology, serum samples were collected from control, T2DM and age-related dementia (ARD) subjects. A significant reduction in serum sCD26 levels was seen in relation to age. ARD and T2DM were also associated with lower levels of sCD26. The analysis of blood smears revealed different cellular morphologies: in controls, CD26 was expressed around the neutrophil membrane, whereas in T2DM, excessive sCD26 was found around the mononucleated cells (MNCs). ARD subjects had abnormal fragmented platelets and haemolysis due to low levels of sCD26.

**Conclusions:** These findings may help to explain the heterogeneity of SARS-CoV-2 infection. High serum sCD26 levels could protect from viral infection by competively inhibiting the virus binding to cellular CD26, whereas low sCD26 levels could increase the risk of infection. If so measuring serum sCD26 level may help to identify individuals at high risk for the COVID-19 infection.

## Introduction

A novel coronavirus, severe acute respiratory syndrome coronavirus2 (SARS-CoV-2) is the cause of the latest pandemic, characterised with severe acute respiratory syndrome (ARS) and high mortality due to both a direct cytotoxic viral effect and a severe systemic inflammation [1–3]. SARS-CoV-2 is a member of the Coronaviridae family, β-coronavirus genus of the B lineage, and is closely related to the severe acute respiratory syndrome coronavirus (SARS-CoV), Middle East respiratory syndrome coronavirus (MERS-CoV), and several bat coronaviruses (CoVs) [4,5]. COVID-19 disease, caused by SARS-CoV-2, has a considerably worse prognosis in older and obese people, some ethnic populations and with comorbidities such as diabetes mellitus, hypertension, cardiovascular disease and chronic lung disease [6–9].

All coronaviruses (CoVs) utilise a portion of the spike protein called the receptor-binding domain (RBD) to recognise and attach to the surface of human cells, allowing the viral particle to gain entry through the plasma membrane [10,11]. SARS-CoV-2 uses angiotensin-converting enzyme-2 (ACE2), the same receptor as SARS-CoV, to infect humans [12]. However, there is recent evidence that the outer membrane spike glycoprotein S1 of SARSviruses binds to dipeptidyl peptidase 4 (DPP4)/cluster of differentiation 26 (CD26) when entering into the cells of the respiratory tract [10,11,13,14]. Differential expression of ACE2 and DPP4/CD26 as receptors of spike glycoproteins may help to explain the heterogeneity of clinical features in people infected with different CoVs [15,16]. For cell entry, SARS-CoV-2 S protein also requires a protease, such as Furin, Trypsin, human airway trypsin-like protease, cathepsin-L or the transmembrane protease serine 2 (TMPRSS2) [16–19]. ACE2 or CD26 may be activated by proteolytic cleavage by TMPRSS2 or cathepsin-L in late endosomes in a pH-dependent manner but the virus can also be activated by trypsin-like proteases at the cell surface in a pH-independent manner [19,20].

DPP4, also known as CD26, is a serine exopeptidase, a multifunctional type-II transmembrane glycoprotein that presents in a dimeric form on the cell surface. CD26 is multifunctional, highly conserved among mammals and plays a major role in glucose metabolism [16]. It preferentially cleaves dipeptides from hormones and chemokines after a proline amino acid residue, thereby regulating their bioactivity [17,18]. CD26, being a proteolytic enzyme that is expressed in most cell types (particularly in the immune cells, T cells and natural killer (NK) cells), exists in both membrane-anchored (mCD26) and soluble form (sCD26) [19]. sCD26 is thought to be shed from the membrane into plasma, and still maintains its enzymatic activity. sCD26 is also found in cerebrospinal fluid (CSF), exerting its multifunctional effects through various signalling pathways that induce and regulate inflammatory and immunological processes [20]. In humans, mCD26 is primarily expressed on lung epithelial cells, endothelial membranes of the pancreas, small intestine, liver, kidney, prostate, placenta and on activated leucocytes [21,22]. Variations in sCD26 levels in serum have been reported as clinically relevant in several pathophysiological conditions including in type 2 diabetes mellitus (T2DM) and virus infections [20]. sCD26 has the capacity to degrade incretins, GLP-1 and glucose-dependent insulinotropic polypeptide (GIP) (both involved in stimulating insulin secretion in a glucose-dependent manner), as well as the brain natriuretic peptide (BNP), and the chemokine stromal-derived factor-1α (SDF-1α), involved in cardiovascular function [23,24].

The emergent global spread of SARS‐CoV‐2 and its grave impact on public health demands an immediate, coordinated effort in biomedical research to increase understanding of the pathogenesis of the virus and its entry into host cells. The high fatality rate imposes an urgent need for rapid identification of effective treatments including novel therapeutics, antivirals and vaccines. We screened mCD26 and sCD26 expression to investigate its role in innate immunity and inflammation [25]. To achieve this, we included samples from T2DM [24] and age-related dementia (ARD) subjects who are considered at high risk for COVID-19 and share some pathological features [26,27]. Via analysis of CD26 involvement in various clinical and pathological conditions that are also characteristic of COVID-19, we provide circumstantial evidence for sCD26 implication in viral infections, such as SARS-CoV-2.

## Materials and methods

### Ethics and participants

Ethics and Research and Development (R&D) approvals were granted by the National Research Ethics Committee of the East of England. Cambridge Health Authorities Joint Ethics Committee granted ethical approval for use of human brain tissue and serum samples (project reference number: REC:15/WM/0379). Written consents were obtained from controls, adults with diabetes participants and subjects with ARD with the capacity to consent. Verbal assent was obtained from participants with ARD lacking capacity to provide written assent, and this was provided instead by an appointed consultee, in accordance with the Mental Capacity Act of the U.K. (2005). Information on older controls (OCs; *n*=50) and ARDs (*n*=50) has already been disclosed in a previous publication [28].

### Paraffin-embedded sections of human brain

Human brain sections of young and aged postmortem CNS-diseased, were provided by the Cambridge Brain Bank under the project reference number REC:15/WM/0379.

### Blood and serum/plasma collection

Whole blood, serum and plasma samples from human younger controls (YCs: *n*=50, aged between 30 and 55 years), and OCs (*n*=50, aged between 56 and 85 years), middle-aged type II diabetes (T2DM, non-demented, *n*=50, aged between 30 and 55 years) (please note that none of the control subjects had a clinical diagnosis of dementia) and ARD (OD, *n*=50, aged between 56 and 85 years) were collected for DNA and protein analyses. All blood samples were collected for serum in BD vacutainer SST advance tubes (containing inert gel barrier and clot activator coating). Serum and plasma were separated immediately by centrifugation at 2465×***g*** for 6 min at 4°C, aliquoted and stored at −80°C until analysis.

### Blood slide preparation

A drop of blood placed on a slide was spread as described previously [29]. The slide was air-dried and fixed in 100% methanol and stored at 4°C. Further investigations, including immunohistochemistry (IHC), were performed on stored fixed slides as described later.

### Animals

Three months old C57/Bl6 mice were purchased from Charles River and bred for the experiments at University of Cambridge Bioscience Facility (UBSS, at John van Geest Centre for Brain Repair Animal Facility). All experiments were performed according to the ‘Animal (Scientific Procedures) Act 1986’ and the ‘Guidance of the Operation of ASPA 2014’, and were approved by the Ethical Committee of the U.K. Home Office (PPL number 70/7920).

### Mice tissue preparation

All animals were housed under standard conditions (12-h light–dark cycle, 20°C ambient temperature) with free access to food and water. Animals undergoing perfusion were anaesthetised with raising concentrations of CO_2_ followed by terminal injection of Euthatal (0.3 ml per 100 g of body weight). Collection of fresh tissues was performed after raising the concentration of CO_2_ followed by cervical dislocation. For histochemical analyses, animals were anaesthetised with pento-barbitone and flash-perfused transcardially with 0.9% saline followed with 4% (v/v) paraformaldehyde (PFA) in 0.1 M phosphate buffer saline (PBS; pH 7.4). Brains were removed, post-fixed for 4 h in the same fixative, and then cryoprotected with 30% sucrose in 0.1 M PBS. Brains and other tissues (spleen and duodenum) were sectioned by microtome as described previously [28]. For *in situ* hybridisation (ISH), unfixed tissues were carefully dissected from C57/Bl6 mice from various brain regions and different organs including spleen, lung, duodenum and embryos (embryonic day 18, ED 18) and snap-frozen in dry ice until analysed by ISH.

### ISH

Brain tissues and other organs were prepared for ISH and probed as described previously [30]. To synthesise DIG-labelled RNA probes, the target CD26 cDNA was amplified by PCR using primers designed on the basis of the mouse CD26 cDNA sequence used in RT-PCR. The primers used for DIG labelling were:

CD26-insF: TAATACGACTCACTATACAAGAAATATCCTCTACTATTAG CD26-insR: ATTTAGGTGACACTATAGAGAAATCCACTTCCAACATCGAC sequenced and homology checked by BLAST search (NCBI database). *In vitro* transcription reactions were performed using dig-UTP RNA labeling mix and SP6 or T7 RNA polymerase (Roche, Mannheim, Germany).

ISH with DIG-labelled probe was carried out on spleen, brain and other embryonic tissues’ sections fixed in 4% PFA for 10 min, permeabilised for 10 min in (0.1 M PBS with 0.5% Triton X-100), and acetylated by 10-min incubation in triethanolamine solution. Pre-hybridisation was performed in hybridisation buffer for 3 h at 62°C and then hybridised with DIG-labelled probes (100 ng/ml) in the same buffer overnight at 62°C. Stringent washing was performed in 0.2× SSC for 1 h at 62°C. For the detection of DIG-labelled hybrids, the slides were equilibrated in maleic acid buffer (MAB, 0.1 M maleic acid and 0.15 M NaCl, pH 7.5), incubated for 1 h at room temperature (RT) with 1% blocking reagent made in MAB (blocking buffer), and then for 1 h with alkaline phosphatase-conjugated anti-DIG antibodies (Roche, Mannheim, Germany) diluted 1:5000 in blocking buffer. The slides were washed twice for 30 min in MAB and incubated overnight in colour development buffer [2.4 mg levamisole (Sigma), 45 μl 4-nitroblue tetrazolium (Sigma), and 35 μl 5-bromo-4-chloro-3-indolyl-phosphate (Sigma) in 10 ml of a buffer made of 0.1 M Trizma base, 0.1 M NaCl and 0.005 M MgCl_2_, pH 9.5]. The reaction was stopped in neutralising buffer (0.01 M Trizma base and 0.001 M EDTA, pH 8) and sections were mounted in PBS/glycerol and a coverslip applied over the section on the slide. Non-specific binding was analysed using sense probes.

### Solid-phase enzyme-linked immunosorbent assay

The sCD26 plasma concentration was measured with the human DPPIV/CD26 DuoSet ELISA development System kit (R&D Systems, Catalogue number DY1180) according to the manufacturer’s instructions. Briefly, for the detection of sCD26, in 96-well culture plates were incubated overnight with monoclonal antibody (mAb) anti-CD26 capture antibody (1 μg/ml) (R&D systems, catalogue number 842127). The following day the plates were washed three times with washing buffer (0.05% Tween 20 in 0.1 M PBS pH 7.4) and blocked in blocking solution (1% BSA and 0.05% Tween 20 in 0.1 M PBS) for 2 h at RT. After blocking, the plates were washed three times with washing buffer and loaded with 10 μl plasma into 90 μl blocking solution and incubated 4 h at RT. All experiments were performed in quadruplicate unless otherwise specified. A recombinant human protein (R&D, Catalogue number 842129) was diluted in assay buffer in a two-fold serial dilution and used for the standard curve with a concentration range of 1000, 500, 250, 125, 62, 31, 15, and 0 pg/ml. After 4 h of incubation, the samples were removed and the plates were washed three times for 5 min with washing buffer before incubation for 2 h at RT for detection with a biotinylated rabbit monoclonal anti-human CD26 (catalogue number 842128, R&D) antibody (1 μg/ml) diluted in blocking buffer. After three further washing steps, the plates were incubated with anti-rabbit HRP-conjugated secondary antibody (1:4000) for 1 h followed by three washes. A total of 100 μl of 1-Step ULTRA tetramethylbenzidine (TMB-ELISA, Thermo Scientific) was added for ∼30 min at RT. Finally, 100 μl of 2 M H_2_SO_4_ was added to quench the reaction. Colorimetric quantification was performed with an Infinite m200 plate reader (Tecan) at 450/540 nm.

### Antibodies

The following primary antibodies were used: mouse monoclonal anti-CD26 (mAb M-A261, Invitrogen) and rabbit polyclonal (pAb) anti-CD26 antibody (Abcam, Ab28340). Other antibodies used in the present study were rabbit pAb anti-apolipoprotein-E (ApoE; Abcam, Ab85311), anti-CD3 (PA5-32318) Thermo Fisher Scientific, anti-cluster of differentiation 42b (CD42b; Abcam, Ab104704), anti-rabbit polyclonal glycophorin (Abcam, 196568), anti-divalent metal protein 1 (DMT1; Abcam, Ab123085), anti-ferroportin (FPN; Abcam Ab85370), anti-GFAP (Abcam Ab48050), anti-CD68 (Sigma–Aldrich, AMAB98073), and anti-CD11b (Thermo Fisher, mAbM1/70). The following secondary antibodies were used: biotinylated goat anti-rabbit-Ig and biotinylated horse anti-mouse (both from Vector Laboratories, 1:250 for IHC); Alexa Fluor 568-labelled donkey anti-mouse-Ig, Alexa Fluor 488-labelled donkey anti-rabbit-Ig, and Alexa Fluor 568-labelled donkey anti-goat-Ig (all from Invitrogen, 1:1000 for immunofluoresence).

### Immunofluorescence

Brain and other sections were blocked using blocking buffer (0.1 M PBS, 0.3% Triton X-100, 10% normal donkey serum) for 1 h at RT, then incubated overnight at 4°C with primary antibody diluted in blocking buffer. Alexa Fluor-conjugated secondary antibodies were used for detection and samples counterstained with 4′,6-diamidino-2-phenylindole (DAPI, Sigma). Sections were then mounted on glass slides with coverslips using Fluoro Save (Calbiochem).

### Microscopy

Bright-field images were taken and quantified using Lucia imaging software and a Leica FW 4000 upright microscope equipped with a SPOT digital camera. Fluorescence images were obtained using a Leica DM6000 wide-field fluorescence microscope equipped with a Leica FX350 camera with ×20 and ×40 objectives. Images were taken through several z-sections and de-convolved using Leica software. A Leica TCS SP2 confocal laser-scanning microscope was used with ×40 and ×63 objectives to acquire high-resolution images.

### Image and statistics analyses

Data were analysed by the paired Student’s *t* test (two-tailed) for two-group comparison, or by ANOVA test for multiple comparison testing. Values in the figures are expressed as mean ± SEM. A one-way ANOVA was used for comparison of data among control, T2DM and ARD and conducted with IBM-SPSS statistic 19 software. Significance was analysed using GraphPad and *P*-values ≤0.001 were considered significant and are indicated in the corresponding figures and figure legends.

## Results

### sCD26 serum protein levels are reduced in dementia, diabetes and in older people

To explore the level of sCD26, serum samples from young and old controls [age-matched T2DM: *n*=50, 30–55 years; non-demented) and ARD (*n*=50, aged between 56 and 85 years) subjects were analysed by sandwich enzyme-linked immunosorbent assay (ELISA) using Human DPP4/CD26 (R&D system, catalogue number: DY1180). All samples were analysed on the same day, using same standard to reduce the day-to-day variation.

**Figure 1:**
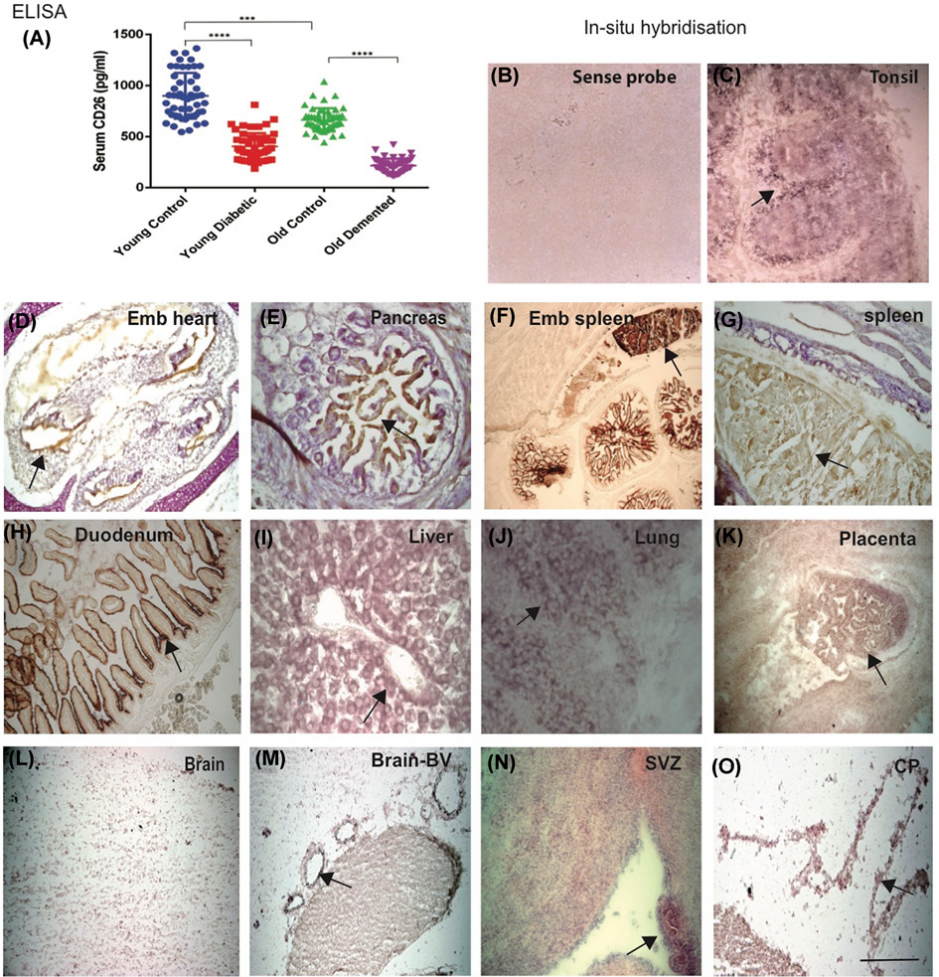
sCD26 serum protein levels are reduced in dementia, diabetes and in older people (**A**) The ELISA analysis performed with human serum showed that CD26 levels were highest in the YCs, followed by OCs, significantly decreased in young diabetics (T2DM) and lowest in the ARD (OD). (YC vs T2DM, and OC vs OD, *P*≤0.0001; YC vs OC, *P*≤0.001 (*P*≤0.001*** and *P*≤0.0001 showed with ****). All serum samples were analysed at the same time to reduce the day-to-day variation. CD26 mRNA expression was detected by ISH using sense and antisense DIG-labelled CD26 probe. A sense probe was used to investigate non-specific binding in tonsil section, no non-specific binding was observed (**B**). Localisation of CD26 enzyme activity in tonsils, a predominant staining of CD26 mRNA was observed in the outer darker extra-follicular T cells region of tonsil with much less staining in the inner germinal centres (**C**), in the endothelium of heart of ED 18 (**D**), endothelial layer of pancreas (**E**), embryonic spleen (**F**), in adult spleen (**G**). In adult mouse duodenum, CD26 expressed in the epithelial brush border (**H**), around the hepatocytes in the liver (**I**), in the lung epithelial cells (**J**) and in the placental trophoblast cells (**K**), (cellular localisation of mRNA indicated by arrow in all panels). There was no mRNA visible in the mouse brain cells (**L**), whereas present in the brain parenchymal blood vessels (**M**), in sub-ventricular zone (SVZ) (**N**) and in the choroid plexus (CP) (**O**). Together, these findings indicated that CD26 mRNA is expressed in T cells, epithelial and endothelial cells of many different organs. Scale bar: (B–F,H–J = 50 μm, K–O = 100 μm).

Levels of sCD26 were lower in older subjects as well as in patients with comorbidities such as T2DM and ARD. The serum protein analysis by ELISA showed that the mean level of sCD26 in control subjects was higher than seen in their older counterparts (901.3 vs 664.2 pg/ml, R^2^ = 0.6, *P*-value ≤0.0001) in YC and OC respectively (Table 1). In younger T2DM, the mean level was (402.9 pg/ml, compared with the mean level in ARD of 216.8 pg/ml, R^2^ = 0.7, *P*-value ≤0.0001) (Figure 1A). A significant reduction in serum sCD26 levels was seen in ARD and in T2DM subjects compared with age-matched controls (Figure 1A and Table 1).

**Table 1:** Soluble Serum CD26 levels in young vs old controls, diabetics and old demented subjects measured by ELISA

Serum CD26 (pg/ml)	YC	Young diabetic	OC	Old demented	
Average (pg/ml)	901.26	402.88	664.22	216.88	
Two-way ANOVA	Ordinary				
Alpha	0.05				
**Source of variation**	**% of total variation**	***P*-value**	***P*-value summary**	**Significant**	
Row factor	5.504	0.6493	ns	No	
Column factor	76.25	<0.0001	****	Yes	
ANOVA table	SS	DF	MS	F (DFn, DFd)	*P*-value
Row factor	970863	49	19814	F (49, 147) = 0.9051	*P*=0.6493
Column factor	13450000	3	4483000	F (3, 147) = 204.8	*P*<0.0001
Residual	3218000	147	21890		
Number of missing values	0				
Tukey’s multiple comparisons test	Mean Diff.	95% CI of diff.	Significant	Summary	Adjusted *P*-value
YC vs. Young diabetic	498.4 (pg/ml)	421.5–575.3 (pg/ml)	Yes		
YC vs. OC	237 (pg/ml)	160.2–313.9 (pg/ml)	Yes	****	<0.0001
YC vs. Old demented	684.4 (pg/ml)	607.5–761.3 (pg/ml)	Yes	****	<0.0001
Young diabetic vs. OC	–261.3 (pg/ml)	−338.2 to −184.4 (pg/ml)	Yes	****	<0.0001
Young diabetic vs. Old demented	186 (pg/ml)	109.1–262.9 (pg/ml)	Yes	****	<0.0001
Old control vs. Old demented	447.3 (pg/ml)	370.4–524.2 (pg/ml)	Yes	****	<0.0001

### CD26 mRNA expression pattern identified in immune cells

Using ISH with a DIG-labelled sense and antisense CD26 probes, we visualised the cellular location of CD26 mRNA in freshly frozen human tonsil, mouse heart, pancreas, embryonic and adult spleen, duodenum, liver, lung and placenta, as shown previously (see ‘Materials and methods’ section) [29]. There was no non-specific binding with sense probe (Figure 1B), whereas with the antisense probe, a predominant staining in the outer darker T cells region of the lymphoid follicle was observed with much less staining in the inner area of the germinal centre (Figure 1C). Higher mRNA expression was seen in the embryonic tissues (in the endothelial layer of the heart, pancreas and spleen) (Figure 1D–F). The highest levels were seen in the placenta, in trophoblast cells, in the spleen, in the duodenum, particularly in the intestinal epithelial cells (Figure 1G–I,K). The membrane-bound mRNA levels was also visible in the lung epithelial cells (Figure 1J). However, very limited mRNA was seen in the mouse brain tissues (Figure 1L), except in blood vessels, sub-ventricular zone (SVZ) and choroid plexus (CP) epithelial cells (Figure 1M–O). Together, these findings indicate that CD26 mRNA is expressed in T cells, epithelial and endothelial cells of many different organs.

### CD26 protein is expressed in the CP epithelial cells and meningeal blood vessels

**Figure 2:**
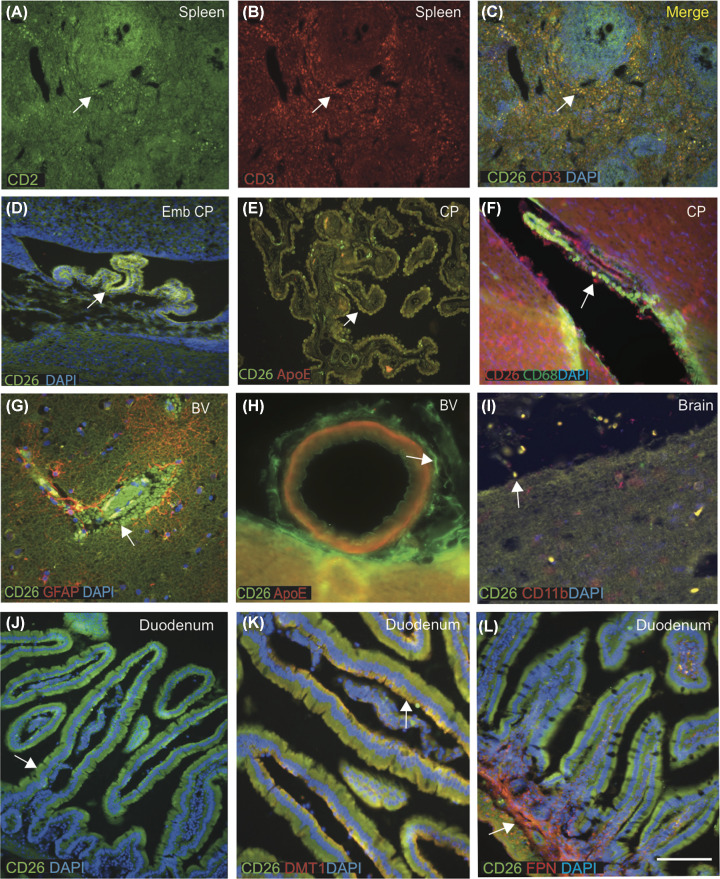
CD26 protein is expressed in the CP epithelial cells, meningeal blood vessels and bind with divalent metal proteins in the duodenum Double IFC staining was performed in the human spleen using mouse mAb anti-CD26 (Green) and rabbit polyclonal (pAb) anti-CD3 (red), and DAPI for nuclei (blue) (**A**–**C**). CD26 immunoreactivity was seen in the human spleen in white pulp, very close to the central canal and in T-cell area surrounding the lymphoid follicles (A,B). CD26 and CD3 (a marker expressed in T cells) colocalised in the outer darker region of the germinal centre containing large- and medium-sized T lymphocytes and both proteins colocalised (C). Embryonic mouse CP was stained with CD26 (green) and DAPI (blue) and imaged using confocal microscopy. Very high CD26 expression was present in the mouse embryonic CP epithelium (**D**). Human normal brain section particularly CP was stained with CD26 (green) and ApoE (red), both proteins were present in the CP epithelial membrane and colocalised in the macrophages (**E**). A mouse brain section from lateral ventricle stained with CD26 (red) and a macrophages marker CD68 (green), CD26-positive macrophages were visible at the wall of ventricle and in the CP, both proteins colocalised (**F**). An ARD brain section from cortex, close to a blood vessel was stained with CD26 (green) and astrocyte marker (GFAP, red), showed that CD26 protein carried by RBCs and macrophages entering through damaged blood vessels (BVs), surrounded by GFAP-positive astrocytes (**G**). Another blood vessel in the brain stained with CD26 (green) visible in the epithelium (**H**). A human brain section from frontal cortex stained with CD26 (red) and a microglial marker CD11b (green) colocalised in the meningeal macrophages in the Pial surface of cortex (**I**). Cellular localisation of CD26 protein indicated with white arrow in all panels). Duodenum sections from adult mouse was stained with CD26 (green) and DAPI (blue), visible in the columnar cells within the crypts (**J**), and colocalised with metal-binding protein DMT1 at the apical surface (**K**). Large populations of T lymphocytes positive for CD26 colocalised with another metal-binding protein FPN at the basolateral site and in the central core of lamina propria containing blood vessels (**L**). Scale bar in (A–F) = 40 μm, (G–L) = 25 μm. Abbreviation: RBC, red blood cell.

COVID-19 infection affects epithelial and endothelial lining of the lung and meningeal macrophages as well as other cells of the immune system [31,32]. For validation of CD26 protein levels in the brain and other immune cells, tissue sections were stained by immunofluorescence (IF) using anti-CD26 mouse mAb (M-A261, Invitrogen) and analysed using confocal microscopy. A clear signal was seen in the human spleen white pulp, very close to the central canal and the T-cell area surrounding the lymphoid follicles (Figure 2A–C). CD26 and CD3 (a marker expressed in T cells) colocalised in the outer darker region of the germinal centre containing large- and medium-sized T lymphocytes (Figure 2C).

There was no visible mRNA expression of CD26 in the adult human or mouse brain, but unexpectedly high levels of proteins were observed in the human CP epithelial cells and meningeal blood vessels (Figure 1M,O). CD26 protein expression was present in the mouse embryonic CP (Figure 2D), human CP epithelial cells and macrophages that colocalised with ApoE (Figure 2E). CD26 protein was seen in the perivascular macrophages and colocalised with CD68 (a known peripheral macrophage marker) in lateral ventricle (Figure 2F). It was visible in the epithelial layer of blood vessels (Figure 2H). When human brain sections (*n*=6) from ARD subjects were stained with CD26 and an astrocyte marker with GFAP antibody, sCD26 protein was detected in the wall of blood vessels: presumably sCD26 which entered the brain parenchyma from blood vessels via astrocytes (Figure 2G,H). CD26 was detected in the meningeal macrophages colocalised with CD11b, a monocyte/microglial marker (Figure 2I).

### Membrane-bound CD26 protein binds with divalent metal proteins in the duodenum

Although SARS-CoV-2 may primarily enter through the lung’s epithelial cells, the small bowel may also be an important entry point or an interaction site [33]. Our *in situ* data (Figure 1H) revealed prominent CD26 expression in the small intestine (duodenum and jejunum). We immunostained mouse gut tissues with CD26 antibody and other known duodenal membrane-binding protein markers, DMT1 and FPN to assess the expression further [34,35]. The most distinct feature of the small intestine is the mucosal lining, with brush border villi and crypts, lined with columnar cells. CD26 staining was seen in the columnar cells within the crypts, particularly in the apical surface and colocalised with metal-binding protein, DMT1, at the apical surface (Figure 2J,K). Large populations of T lymphocytes positive for CD26 colocalised with another metal-binding protein (FPN) at the basolateral site and in the central core of lamina propria containing blood vessels (Figure 2L). Throughout the small intestine, confluent lymphoid tissues known as Peyer’s patches were visible and CD26 positive (Figure 2L).

### CD26 and coagulopathy

**Figure 3:**
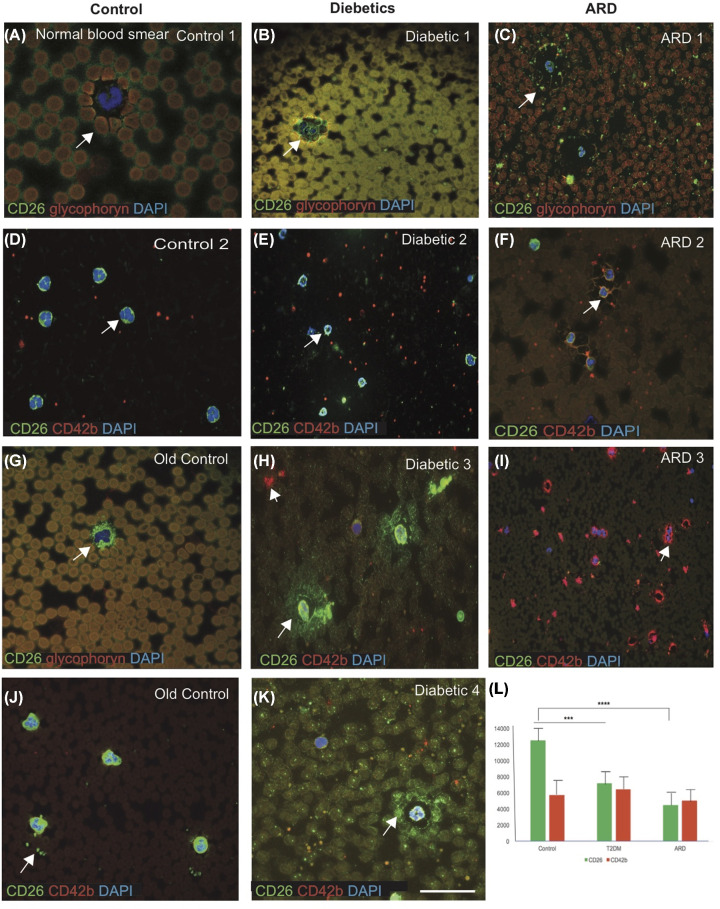
CD26 is expressed on the surface of MNCs around the outer membrane, and a web-like appearance that colocalised with integrin observed The blood smears from normal, T2DM and ARD were stained with CD26 (green) and either glycophorin (red) or CD42b (red) and imaged using confocal microscopy. In normal blood smears, glycophorin was expressed on the surface of RBCs (**A**) and CD26 seen around the RBCs in T2DM and ARD (**B,C**). Blood smear showed normal MNCs surrounded by CD26 (green) and CD42b (red) in normal platelets (red) (**D**), in T2DM (**E**) and in ARD (**F**). In T2DM, there was excessive sCD26 around the MNCs and some clumped platelets (red) in the periphery (**H**–**K**). Demented (ARD) subjects showed a web-like expression of CD26 proteins (green) present surrounding the neutrophil and granulocytes (**F**), and fractured platelets (red) around the MNCs with visible nets of integrin (**I**). One old control blood smear stained either with CD26 (green) and either with glycophoryn or CD42b (red). CD26 was present around the MNCs (**G**) and some platelets colocalised with CD26 and CD42b (**J**). Cellular localisation of CD26 protein indicated with white arrow in all panels). CD26 level was highest in the controls, lower in T2DM and lowest in the ARD (**L**). Scale bar in A–K = 10 μm. ***P≤0.001, ****P≤0.0001.

One of the emerging hallmarks of COVID-19 is a coagulopathy, termed as ‘sepsis-induced coagulopathy’ (SIC) with elevated D-dimer and fibrinogen levels [36]. It is related to an infection-induced systemic inflammatory response with endothelial dysfunction and microthrombosis often leading to organ failure [36–38]. Several observational studies have characterised monocytes during SARS-CoV-2 infection [39]. We investigated cellular expression of CD26 in blood smears from different age groups (*n*=20, age between 30 and 85 years) and imaged using confocal microscopy. The blood smears from young and old subjects were co-labelled with CD26 monoclonal antibody and either with an erythrocyte plasma membrane marker (glycophorin) or a platelet marker (CD42b). Glycophorin was expressed on the surface of red blood cells (RBCs) (Figure 3A–C) with CD26 seen around the RBCs (could be on erythrocytes spikes) (Figure 3A,B). There was stronger CD26 expression around the RBCs membranes in the controls (Figure 3A,G) and in T2DM compared with ARD (Figure 3B,C). CD26 was expressed on the surface of mononucleated cells (MNCs) around the outer membrane (Figure 3D,E), and a web-like appearance that colocalised with integrin CD42b protein was observed (Figure 3F). The control subjects had stronger CD26 expression around the neutrophil membrane (Figure 3D,G,J), whereas in T2DM there was excessive sCD26 around the MNCs and haemolysis in RBCs (Figure 3H,K). The young and old control subjects expressed much more CD26 in MNC (Figure 3D,G,J,L), whereas younger T2DM and older ARD subjects carried abnormal fragmented platelets stained with CD42b (Figure 3H,I). Additionally, scattered sCD26 was observed extracorporally of RBCs and within MNCs in the T2DM samples (Figure 3H–K). A classic dysmorphology (bilobed neutrophils) was frequently seen in blood of T2DM (Figure 3H,K) whereas normal neutrophils positive for CD26 were found in controls (Figure 3D–G).

In ARD subjects (with dementia), RBCs appeared abnormally shaped (macrocytic with damaged erythrocytes spikes) and platelets found were fragmented (Figure 3F–I). These findings suggest that further investigation of CD26 involvement in lymphocyte regulation is warranted, especially in relation to the coagulation cascade.

## Discussion

COVID-19 caused by SARS-CoV-2 is now a pandemic [2,3,40]. SARS-CoV-2 pathology is similar to that of SARS-CoV- and MERS-CoV-induced viral pneumonia [41]. CoV has specific immune response and immune escape characteristics and can cause severe pathogenic mechanisms through inflammation leading to a variety of symptoms like acute respiratory distress syndrome, gastrointestinal disorder, renal failure, vascular bleeds, stroke, T cell-specific immune responses and cytokine storm [4,42,43]. Cytokine storm is hypercytokinemia that increases the volume of pro-inflammatory cytokines in the serum (e.g. IL-1β, IL-6, IL-12, INF-γ) and chemokines (CXCL10 and CCL2), and is correlated with pulmonary inflammation and extensive lung involvement as seen in COVID-19 patients [44]. MERS-CoV infection was also described to provoke increased concentration of cytokines (IL-15, IL-17, TNFα and INF-γ) [45]. It is reported that victims infected with SARS-CoV-2 also demonstrate high levels of IL-1β, TNFα, INF-γ, CXCL10 and CCL2 which may be attributed to the activated response of T helper 1 (Th1) cells [46]. CXCL10 binds to CXCR3, a G-protein-coupled receptor, which is known to be expressed on activated T lymphocytes, NK cells and some epithelial and endothelial cells. CXCL10–CXCR3 acts in an autocrine fashion on the oxidative burst and chemotaxis in the inflamed neutrophils, leading to fulminant pulmonary inflammation [47].

Recently, sequence and modelling analyses of S glycoproteins of MERS-CoV, SARS-CoV-2 and further co-purification with the MERS-CoV S1 domain, identified that CD26 functions as a cellular receptor for MERS-CoV and possibly SARS-CoV-2 [16,48]. Another recent study reported a correlation between CD26 and ACE2, suggesting that both membrane proteins could be relevant in the pathogenesis of virus entry [49]. The co-expression of ACE2 and CD26 as receptors of spike glycoproteins suggests that different human CoVs may target similar cell types across different human tissues, and this may also explain the presence of similar clinical features in patients infected with different CoVs. In addition, it was shown that CD26 acted as a CoV co-receptor, thus suggesting a potential similar mechanism of entry for SARS-CoV-2. There is a connection between the SARS-CoV, SARS-CoV-2, ACE2 and the rationale for soluble ACE2 as a potential therapy by using ACE2^+^-small extracellular vesicles (sEVs) [50]. We suggest that soluble CD26 circulates via platelets and T cells carrying vesicles as shown in the normal blood smear (Figure 3).T herapeutic approaches may be envisaged using sCD26 as a decoy receptor which could inhibit the entry of the virus through cell surface [16,50,51]. In this study, we analysed the expression pattern in healthy subjects and subjects who are at high risk for COVID-19. Our findings now need to be validated with COVID-19 patients.

The role of CD26 in immune regulation has been extensively characterised, with findings elucidating its linkage with signalling pathways and structures involved in T-lymphocyte activation as well as antigen presenting dendritic T-cell interaction [52,53]. In this study, we show that CD26 is expressed in T cells, the extrafollicular area of tonsil and at T-cell areas in the germinal centre of the spleen and is also colocalised with CD3 [54]. The outer darker region of the germinal centre of tonsil, showed that it contained large- and medium-sized lymphocytes as described before [54]. Spleen may be one of the organs directly attacked by the virus in some patients who died from COVID-19. T and B lymphocytes in the spleen decreased in varying degrees, lymphoid follicles are atrophied, decreased or absent [55]. T cells secrete cytokines and chemokines in immune cells as reported in some genes present in MHC region [56]. These findings suggest that the binding of sCD26 to the spike (S) glycoprotein may also depend on T-cell population.

Although SARS-CoV-2 may primarily enter the cells of the lungs, the small bowel may also be an important site of entry or interaction site, as the enterocytes are rich in ACE2 receptors [33]. Duodenal mucosa expresses mCD26 particularly on the apical site of the brush border epithelial cells where T cells present and bind with many divalent metals including Fe^+^, Ca^+^, Co^+^, Zn^+^ and Hg^+^ [57]. Particularly, iron (Fe^+^) plays an important role in inflammation and causes disbalance and hyperferritinaemia in COVID-19 [58]. Most iron absorption occurs in the duodenum via the DMT1-mediated uptake and FPN1-mediated export across the apical and basolateral membranes respectively [34,35]. CD26 is a cysteine-rich protein, that can bind with divalent metal-binding proteins (DMT1) and FPN [28,34,35]. Much of this research focus has centred on the ectodomain of the spike protein. The ectodomain is anchored to a transmembrane region, followed by a cytoplasmic tail. Recently, it was reported that a sequence similarity exists between the cysteine-rich cytoplasmic tail of the CoV spike protein with the cysteine-rich domain in hepcidin protein, which is a key regulator of iron homoeostasis in humans and other vertebrates has been reported [59–61]. SARS-CoV-2 spike (S) glycoprotein can bind DMT1 on the apical site of brush border epithelial cells in the gut mucosa, and could mediate membrane fusion and virus entry into the cells. High levels of sCD26 can therefore potentially compete and prevent viral entry through gut mucosa and exert beneficial immunoprotective effects via elevation of GIP, a peptide that is produced in gastrointestinal K cells and further proteolysed by CD26 [62]. As a consequence, the small bowel may serve as a viral entry site or as a potentiating organ, magnifying the systemic inflammatory response, small bowel being the largest lymphoid organ of the body. A significant proportion of COVID-19 patients with gastrointestinal symptoms clearly support the involvement of the small bowel in SARS-CoV-2 [33].

One of the emerging hallmarks of COVID-19 is a coagulopathy, termed as ‘SIC’ with elevated D-dimer and fibrinogen levels [36]. It was shown that platelet activation and platelet–leucocyte interactions participate in the pathophysiology of viral infections, including dengue, HIV and influenza [63]. Recently, a hypercoagulability state has been reported as a major pathologic event in COVID-19, and thromboembolic complications are listed among life-threatening complications of the disease [64]. Platelets are chief effector cells of haemostasis and pathological thrombosis [65]. In this paper, we demonstrated that increased platelet activation and platelet–monocyte aggregate formation are observed in diabetic subjects (Figure 3E,H), and much more prominent in ARD (Figure 3F,I) but not in controls (Figure 3D,G).

Recent clinical evidence has revealed T2DM and cardiovascular risk factors increase susceptibility to COVID-19 infection [66]. The elderly, and those with underlying medical conditions particularly obesity, diabetes, ARD are the pre-existing diseases associated with the greatest risk and death in COVID-19 pandemic [26,27,67]. We therefore chose T2DM, ARD and age-matched young and old controls who are at high risk for COVID-19 infection. They all could thus have common risk factors that affect different organs, particularly the epithelial membrane responsible for the viral entry. We found that CD26 is expressed in the epithelial membrane of the CP and in the endothelium of blood vessels in the brain. The CP is a secretory tissue responsible for producing both the CSF in the vertebrate brain and many innate immune molecules to protect the brain from infection by surveying the blood–brain barrier (BBB) [29,68]. CD26 protein is present in the CP epithelial membrane and in the outer layer of brain pia mater particularly in the meningeal macrophages, where it is colocalised with other brain spectrins, known as fordin and ankyrin [29,69]. Similarly, CD26 is expressed in erythrocyte membranes (also having erythrocyte spikes) and is colocalised with membrane proteins, glycophorin and integrin α2β3 [70]. Since SARS-CoV-2 spike (S) glycoprotein binds to erythrocytes, causing clotting defects in the small blood vessels [37,71], we analysed blood smears from young, diabetic (T2DM) and older subjects (ARD). In young controls, the higher levels of sCD26 around neutrophils, and in platelets was seen, whereas in immunocompromised T2DM subjects many fractured platelets and clumped RBCs with haemolysis were visible, alongside a web-like expression of integrin proteins was present surrounding the neutrophil and granulocytes (also visible in ARD subjects). Furthermore, in ARD subjects macrocytic, broken RBCs and fragmented platelets were present. These findings demonstrated the impaired coagulopathy in older subjects and those with T2DM and dementia, and complement our previous findings of another immunoprotective molecules such as TREM2, suggesting that CD26 may also be required for the haemopoietic cellular growth and protection [72].

Levels of serum CD26 are approximately three-times higher in pregnant women at ∼3 μg/ml (R.R.-C., unpublished data). CD26 mRNA expression is elevated in placental trophoblasts, where it may induce regulatory T cell (Treg) differentiation and protect the foetus from external infections [73]. These findings suggest that newborn babies and young children may be protected from COVID-19 due to high levels of sCD26. Another study reported female HIV positive but disease-free sex-workers in Nairobi also showed higher levels of serum sCD26 and thereby presumably protecting them from all other viral infections [74]. Our findings suggest that T-cell regulatory CD26 is expressed in different organs from tonsil, spleen, gut mucosa to brain (particularly in CP and in BBB) and sCD26 may play a crucial role as a decoy receptor which could inhibit the entry of the virus through cell surface, allowing more time for neutralising antibodies to be raised against the SARS-CoV-2 spike protein.

## Conclusions

We have shown sCD26 expressed in serum, and mCD26 in T cells (in tonsil and spleen), in CP, duodenum and erythrocytes. We observed a significant reduction in serum sCD26 levels with age and in T2DM subjects compared with age-matched controls. Thus, we suggest that measuring sCD26 level in the serum of young and old control subjects and comparing them with SARS-CoV-2 virus carrying subjects could be explored as a possible test for inflammatory stages in COVID-19: high serum sCD26 level could protect from viral infection by blocking the receptor from virus entry, whereas low sCD26 level may be associated with a higher risk of infection. If correct,a therapeutic approach may be to use sCD26 as a decoy receptor which could competitively inhibit the entry of the virus through cell surface.

## Perspectives

- The rapid global spread of SARS‐CoV‐2 and its grave impact on public health immediately demands prompt and efficient coordinated effort in biomedical research to increase the understanding of the pathogenesis of the virus and its impact upon the host cells. T2DM and cardiovascular risk factors increase the susceptibility to COVID-19 infection. Variations in sCD26 levels in serum have been reported as clinically relevant in several pathophysiological conditions including T2DM and virus infections, where CD26 may plays a critical role in the innate immunity and T-cell regulation.
- We investigated the mCD26 and sCD26 expression to uncover CD26 role in the innate immunity and inflammation. For this, we analysed samples from T2DM and ARD subjects who are considered at high risk for COVID-19. We show sCD26 is expressed in serum, T cells (in tonsil and spleen) whereas mCD26 in CP, duodenum and erythrocytes.
- We suggest that measuring sCD26 level in the serum should be explored as a possible test for inflammatory stages in COVID-19: high serum sCD26 level could protect from viral infection by blocking the receptor from virus entry, whereas low sCD26 level may be associated with a higher risk of infection. It could be theraputic approches to use sCD26 as a decoy receptor which could competitively inhibit the entry of the virus through cell surface.

## Data Availability

All data analysed in the present paper are already included in the manuscript, including a table.

## Open Access

Open access for this article was enabled by the participation of University of Cambridge in an all-inclusive *Read & Publish* pilot with Portland Press and the Biochemical Society under a transformative agreement with JISC.

## Abbreviations

ACE2: angiotensin-converting enzyme-2
ApoE: apolipoprotein-E
ARD: age-related dementia
ARS: acute respiratory syndrome
BBB: blood–brain barrier
CCL2: C-C Motif Chemokine Ligand 2
CD26: cluster of differentiation 26
CD42b: cluster of differentiation 42b
CNS: Central nervous system
CoV: Coronavirus
COVID-19: Coronavirus-19
CP: choroid plexus
CSF: cerebrospinal fluid
CXCL10: C-X-C motif chemokine ligand 10
DMT1: divalent metal protein 1
DPP4: dipeptidyl peptidase 4
ELISA: enzyme-linked immunosorbent assay
FPN: ferroportin
GFAP: Glial fibrillary protein
GIP: glucose-dependent insulinotropic polypeptide
IF: immunofluorescence
IHC: immunohistochemistry
INF: Interferon
ISH: *in situ* hybridisation
MAB: maleic acid buffer
mAb: mouse monoclonal antibody
mCD26: membrane-anchored CD26
MERS-CoV: Middle East respiratory syndrome coronavirus
MNC: mononucleated cell
NK: natural killer
OC: older control
PBS: phosphate buffer saline
PFA: paraformaldehyde
RBC: red blood cell
RT: room temperature
SARS-CoV: severe acute respiratory syndrome coronavirus
SARS-CoV-2: severe acute respiratory syndrome coronavirus-2
sCD26: soluble CD26
SIC: sepsis-induced coagulopathy
T2DM: type 2 diabetes mellitus
TMPRSS2: transmembrane protease serine 2
TREM2: Triggering receptor expressed on myeloid cells 2
Treg: regulatory T cell
YC: younger control
